# Proteomics research in forest trees: A 2012-2022 update

**DOI:** 10.3389/fpls.2023.1130665

**Published:** 2023-04-05

**Authors:** María Angeles Castillejo, Jesús Pascual, Jesus V. Jorrín-Novo, Tiago Santana Balbuena

**Affiliations:** ^1^ Agroforestry and Plant Biochemistry, Proteomics and Systems Biology, Department of Biochemistry and Molecular Biology, University of Cordoba, Cordoba, Spain; ^2^ Plant Physiology, Department of Organisms and Systems Biology, University of Oviedo, Oviedo, Spain; ^3^ University Institute of Biotechnology of Asturias, University of Oviedo, Oviedo, Spain; ^4^ Department of Agricultural, Livestock and Environmental Biotechnology, School of Agriculture and Veterinary Sciences, São Paulo State University (UNESP), Jaboticabal, São Paulo, Brazil

**Keywords:** forest, tree, proteomics, eucalyptus, pinus, quercus

## Abstract

This review is a compilation of proteomic studies on forest tree species published in the last decade (2012-2022), mostly focused on the most investigated species, including *Eucalyptus, Pinus*, and *Quercus*. Improvements in equipment, platforms, and methods in addition to the increasing availability of genomic data have favored the biological knowledge of these species at the molecular, organismal, and community levels. Integration of proteomics with physiological, biochemical and other large-scale omics in the direction of the Systems Biology, will provide a comprehensive understanding of different biological processes, from growth and development to responses to biotic and abiotic stresses. As main issue we envisage that proteomics in long-living plants will thrive light on the plant responses and resilience to global climate change, contributing to climate mitigation strategies and molecular breeding programs. Proteomics not only will provide a molecular knowledge of the mechanisms of resilience to either biotic or abiotic stresses, but also will allow the identification on key gene products and its interaction. Proteomics research has also a translational character being applied to the characterization of the variability and biodiversity, as well as to wood and non-wood derived products, traceability, allergen and bioactive peptides identification, among others. Even thought, the full potential of proteomics is far from being fully exploited in forest tree research, with PTMs and interactomics being reserved to plant model systems. The most outstanding achievements in forest tree proteomics in the last decade as well as prospects are discussed.

## 1 Introduction

Forest species are important from an ecological, social, and economic point of view. The total forest area covers 4.06 billion hectares around the world (https://www.fao.org/state-of-forests/en/), contributing to the soil and water resources conservation, global carbon uptake and storage and is largely responsible for the consistency of the global carbon sink (Hurteau, 2021). Besides, forests harbor much of the world’s terrestrial biodiversity and are an inexhaustible source of pharmaceuticals, and other important wood and food derived goods to people. Deforestation and forest degradation threaten the survival of many species and reduce the ability of forests to provide goods and essential services. Among the main causes behind the loss of forests are pathogens attack, forest fires, and others of anthropogenic origins. Halting the loss and degradation of forest ecosystems and promoting their restoration to mitigate global climate change are among the main objectives of scientists for the 2030 Paris Agreement on climate change. We quote the recent message from the FAO on the current situation of the forests: “Trees, forests, and sustainable forestry can help the world combat looming environmental crises such as climate change and biodiversity loss” (FAO, 2022).

In this sense, research about the biology of forest species, life cycle, biodiversity, and responses to environmental stresses, among others, will help predict how these species will behave in a context of climate change, for the selection of the best-adapted species or individuals that can be used in afforestation and reforestation programs, as well as to the natural regeneration of forest ecosystems. Despite its importance, research in forest trees is underrepresented compared to other plant species, such as crops. Thus, a Pubmed (https://pubmed.ncbi.nlm.nih.gov) search covering the period 2012-2022 returned 9,846 hits using as a search string “forest-tree” (3,248 for *Eucalyptus*, 4,759 for *Pinus*, and 2,733 for *Quercus*), and 50,325 using “crop”. Molecular research in forest species is even more limited, mainly caused by the high genetic variability, longevity, long regeneration periods, allogamy, seed recalcitrance, and lack of genomic tools (Rodrigues et al., 2021; Maldonado-Alconada et al., 2022). Previous reviews have already been published collecting works carried out on forest species at the molecular level, focused on the species (Rey et al., 2019; Escandón et al., 2021a; Maldonado-Alconada et al., 2022), the stress (Naidoo et al., 2019; Rey et al., 2019; Amaral et al., 2022; Modesto et al., 2022) or the technique used (Abril et al., 2011; Du et al., 2018; Rey et al., 2019; Rodrigues et al., 2021).

Most of the forest tree proteomic works aimed to study biological processes such as growth and development, responses to stress, prototyping, and the characterization of natural variability for the identification of proteins that could be used as markers in breeding programs. Lately, proteomics has been applied to the study of seed recalcitrance (Romero-Rodríguez et al., 2019; Sghaier-Hammami et al., 2021; Escandón et al., 2022) and traceability, proving the nutraceutical value of seeds and derived products, with a clear translational potential in relation to the identification of allergens and bioactive peptides (Pedrosa et al., 2020; Maldonado-Alconada et al., 2022). The development of techniques and databases of non-model organisms has meant a qualitative leap for the advancement of proteomics in forest species.

In the last 10 years, proteomics techniques moved from gel-based strategies to gel-free shotgun and lately to targeted approaches. This mini-review attempts to compile proteomic research articles and reviews published in the last decade focusing on the genera *Quercus*, *Pinus*, and *Eucalyptus* with an emphasis on the species of the authors’ research work. The relevance as a biological system and the methodology used is reviewed in sections devoted to each species. **Table S1** provides a list of the main proteomics works addressed in the last 10 years, which describes the species, organ/tissue, objective, methodological strategy used and the main results obtained.

## 2 Proteomics survey (2012-2022): Where are we now?

**Figure 1:**
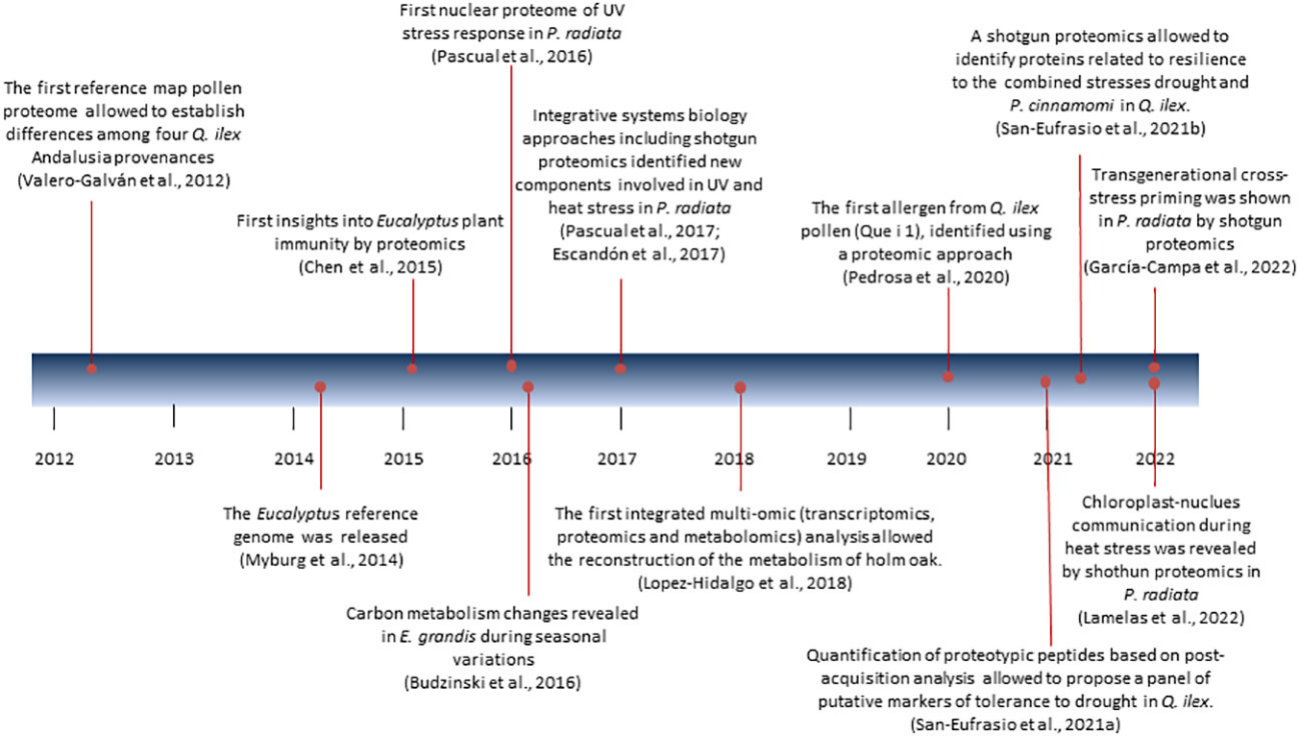
Main milestone in research on forest species in the last decade.

In the last decade we have witnessed a gradual transition from the use of gel-based to gel-free techniques in plant proteomics, which are often combined. Two-dimensional gel electrophoresis (2DE) coupled to MALDI-TOF is the star proteomics technique that undoubtedly has been used until 2016-2017 in Eucalyptus and Pine, and a bit later (2019) for Holm oak. As of 2017 in the first two, and as of 2020 in the last one is when the use of gel-free techniques (LC-MSMS) DDA and DIA based, or targeted was chosen. Next, a tour of the greatest achievements in proteomics and the techniques used in each of these species is presented (**Figure 1**; **Supplemental Table S1**).

### 2.1 *Eucalyptus* spp.

*Eucalyptus* are fast-growing trees comprising approximately 700 species and hundreds of different commercial hybrids. Native to Australia, Indonesia, the Philippines, and New Guinea (Paine et al., 2011), *Eucalyptus* plants were successfully introduced in several countries, where they are mostly used for the pulp and paper industry. Currently, the total *Eucalyptus* plantation area exceeds 22 million hectares worldwide (Zhang and Wang, 2021). Understanding the biochemical and molecular basis that leads to plant growth and adaptation may contribute to the enormous challenges posed by future climate scenarios. Proteome differences in two ecophysiologically different *Eucalyptus* genotypes were investigated in field conditions to identify metabolic changes induced by water stress using a 2DE coupled LC-MSMS approach (Bedon et al., 2012). The same strategy was employed to study adaptative responses to drought stress in seeds from native populations with contrasting drought sensitivities in *E. globulus* (Valdés et al., 2013) and plantlets of *E. saligna* and *E. tereticornis* for water stress (Martins et al., 2020). From all environmental responses studied in *Eucalyptus* plants to our knowledge, the thermal stimulus appears to be the most representative one, although none of those studies was carried out in field trials. Finding potential molecular markers and studying global metabolic changes induced by alterations in the growth temperature was investigated in several different *Eucalyptus* species using mainly gel-free strategies (Aspinwall et al., 2019; de Santana Costa et al., 2017; Costa et al., 2020, Leonardi et al., 2015; Oberschelp et al., 2020). Chloroplast proteome was also investigated in *E. urophylla* in order to identify changes in the abundance of Calvin-Benson and antioxidant enzymes induced by growth in CO_2_ enriched atmosphere using a GeLC-MSMS analysis (Santos and Balbuena, 2017; Baldassi and Balbuena, 2022). In face of the climate change challenge, proteomics can be used to predict real changes from experimentally induced scenarios. Since most climate predictions were confirmed in the last decades, investigating the proteome changes induced by combined stimulus appears to be the right choice for screening more realistic responses. Recently, Correia et al. (2018) aimed at mimicking a more realistic future scenario by challenging *E. globulus* plantlets against combined drought and heat stress.

Understanding how *Eucalyptus* species interact with the environment opens new biotechnological perspectives for these perennial plants, such as bioremediation. It has been observed that *E. camaldulensis* plants have the potential to phytoremediate cupper-specific and heavy metal contaminated sites (Guarino et al., 2014; Alotaibi et al., 2019). Using gel-based proteomics differentially abundant proteins were suggested as key molecules in the formation of chelating complexes; however, the causal relationship is still to be defined. Plant-pathogen interaction is another theme of great importance for *Eucalyptus* plantations. Multi-omics approaches have been used to understand the defense response in *E. grandis* against rust infection (Sekiya et al., 2021) and *Calonectria pseudoreteaudii* (Chen et al., 2015), the biological agent of the *Calonectria* leaf blight disease. Although strictly descriptive, these studies provide the molecular basis for a future mechanistic overview and functional characterization of the molecular players involved in pathogen-related responses in *Eucalyptus*. Seasonal variations (Budzinski et al., 2016a; Budzinski et al., 2016b; Baldassi and Balbuena, 2022), direct agricultural application in intercropping systems (Yao et al., 2021), and cellular signaling (Plett et al., 2017) were also investigated in the last decade from different approaches, LC-MSMS, targeted (Parallel Reaction Monitoring) proteomics, independent or combined with transcriptomics or metabolomics analysis. Last but not least, a proteogenomics approach, refers to the strategy of searching for peptide identities derived from spectrometric data using custom databases, and *de novo* peptide sequencing analysis was recently used for the identification of novel protein-coding sequences in the *E. grandis* genome (Jorge and Balbuena, 2021), paving the way for a more comprehensive overview of the *Eucalyptus* proteome through a combinatorial bioinformatics data mining approach.

### 2.2 *Pinus* spp.

The *Pinus* genus includes 187 species (http://www.worldfloraonline.org/) most commonly found in the northern hemisphere. *Pinus* are fast-growing trees widely used for several economical purposes, timber production being one of the main ones. Pines account for 29.6% of the growing stock in European forests. Moreover, around one-third of European forests are dominated by a single tree species, very commonly a pine (Forest Europe, 2020). Therefore, pine species have great ecological and economical value, and understanding the molecular mechanisms governing development and adaptation is of great interest. In this regard, the contribution of proteomics has been remarkable (Jorrín-Novo et al., 2015). The last decade has witnessed a huge advance in pine proteomics research that has continued the transition from gel-based to gel-free systems based on mass spectrometry thanks to important methodological developments, such as those of databases (Nystedt et al., 2013; Zimin et al., 2014; Stevens et al., 2016; Zimin et al., 2017) and their use (Romero-Rodríguez et al., 2014). In addition, the development of new protein isolation protocols (Valledor et al., 2014; Colina et al., 2020) and fractionation methods has allowed studying subcellular proteomes (Alegre et al., 2016; Lamelas et al., 2020a). Noteworthy, proteomic analysis has been very commonly performed in combination with other omics, like transcriptomics or metabolomics, which has needed new computational strategies and algorithms to allow the integration of different omic layers effectively, and comprehensively (Escandón et al., 2020; Sundararaman et al., 2020).

Proteomics has been especially used for studying stress response (**Supplemental Table S1**). Abiotic stress studies have aimed at mimicking near-future conditions pines will face because of the ongoing climate change to get insight into their adaptation capacity. Acid rain and especially UV and heat stress have been intensively studied. Proteomics by 2DE coupled to MALDI-TOF/TOF has been used to study the effects of acid rain in *P. massoniana* (Hu et al., 2014a) and the role of calcium in the stress response it induces (Hu et al., 2014b). An integrated physiological, proteomic, and metabolomic analysis of *P. radiata* seedling needles’ response to UV and recovery using shotgun proteomics revealed a remodeling of the proteome associated with metabolism rearrangement to deal with oxidative stress (Pascual et al., 2017). Protein kinases and proteases were associated with signaling and regulatory processes during the UV stress response. A complementary study, analyzing changes in the needle nuclear proteome by shotgun proteomics, identified the main transcription factor families governing UV stress response (Pascual et al., 2016). A similar approach was also used in a system-wide analysis of short-term response to high temperatures in *P. radiata* seedlings (Escandón et al., 2018). This approach uncovered the importance of proteins related to hormone signaling and lipid and flavonoid metabolism. Phosphate transporter 1 (PHO1) and the transcription factor APFI were identified as potential heat-stress resistance biomarkers. The accumulation of small heat shock proteins (sHSPs) was also reported. The response to prolonged heat stress was also studied in *P. radiata* at the nuclear proteome level (Lamelas et al., 2020b). The use of a two-phase stress experimental design further confirmed the importance of sHSPs after initial heat stress and found changes in activated methyl cycle enzymes and H2A-H2B histone dimers associated with stress memory. Lamelas et al. (2020b) also described changes in spliceosome-related proteins during heat stress and recovery, suggesting alternative splicing as an important mechanism mediating stress response and memory, which was further characterized by Roces et al. (2022). Furthermore, the combined study of nuclear and chloroplast proteomes revealed the importance of proteins related to retrograde and anterograde signaling and to RNA metabolism rearrangement mediated by microRNAs, revealing a new layer of regulation in heat stress response (Lamelas et al., 2022). Proteomic studies on heat stress in somatic embryos have found some commonalities in the proteins and processes involved, opening the door to the production of thermo-primed plants (Castander-Olarieta, 2021; Castander-Olarieta et al., 2022).

Most recent studies have used genetic variation-based approaches to study stress memory and cross-tolerance, and to define stress resistance markers. Baniulis et al. (2020) identified constitutive differences in protein abundance associated with cold acclimation capacity in different *P. sylvestris* populations. García-Campa et al. (2022) identified proteins related to photorespiration, redox homeostasis, and secondary metabolism associated with transgenerational stress cross-tolerance and priming analyzing the chloroplast proteome of the progeny of two *P. radiata* populations with the same genetic background but from environmentally contrasting locations.

Biotic stress research has also used genetic variance and proteomics aiming in this case at identifying the proteins responsible for resistance/susceptibility to two main pathogens affecting pine species worldwide currently: the fungus *Fusarium circinatum*, responsible for the pine pitch canker (Wingfield et al., 2008; Amaral et al., 2021; Amaral et al., 2022), and the pine wood nematode *Bursaphelencus xylophilus*, causing pine wilt (Espada et al., 2022). Comparative proteome analysis of the differential response of pine species upon *F. cicinatum* inoculation revealed that susceptibility was associated with proteins involved in negative regulation of plant immunity, and increased energy production and amino acid synthesis pathways related to changes in plant secondary metabolism and chloroplast redox balance. In turn, proteins related to vesicle trafficking and the crosstalk between ABA and epigenetic regulation were associated with pathogen resistance (Amaral et al., 2021). Similar approaches have been used to study the interaction between pine species and the pine wood nematode, *B. xylophilus*. Proteomics has been used to characterize the nematode secretome. These works have revealed the importance of peptidases, hydrolases, and antioxidant proteins in overcoming the defense mechanisms deployed by different hosts (Silva et al., 2021; Cardoso et al., 2022). A recent study used Masson pine (*P. massoniana*) clones selected through traditional breeding over 20 years and screened for different resistance to pine wilt (Gao et al., 2022). Comparative Tandem Mass Tagged (TMT) based quantitative proteomic analysis combined with parallel reaction monitoring (PRM) identified proteins related to SA metabolism, the antioxidant system, polysaccharide degradation, and lipid biosynthesis to change significantly during the infestation process. This study showed that the capacity of the plant to degrade nematode-related proteins and to downregulate its carbon metabolism to limit carbon availability for the nematode might diminish the infestation capacity of the nematode.

### 2.3 *Quercus* spp.

*Quercus* genus (family Fagaceae) includes 464 spp. distributed throughout the Northern Hemisphere to Malaysia and Colombia (Kew Royal Botanic Garden: https://powo.science.kew.org/results?q=Quercus). Playing an important role in human life since the prehistoric period, oaks are the most important woody species in terms of diversity, ecological dominance, and economic value, being a source of a wide variety of goods and services for humans and animals (Leroy et al., 2020; Backs and Ashley, 2021). Among them *Q. suber* and *Q. ilex* are the dominant species in natural forest ecosystems over a large area of the Western Mediterranean Basin, and in the agrosilvopastoral Spanish “dehesa” (Olea and San Miguel-Ayanz, 2006; De Rigo and Gaudullo, 2016; Surová et al., 2017). With a high ecological, social, and economic value due to the cork, for the former, and acorns, for the latter, these species have been the best characterized at the proteomic level (Abril et al., 2011; Ricardo et al., 2011; Rey et al., 2019; Maldonado-Alconada et al., 2022; Saiz-Fernández et al., 2022). In the case of *Q. ilex*, there is also a current and renewed interest in the use of acorns for dietary diversification and sustainable food production. However, the survival of these species is threatened by various anthropogenic and environmental factors, among which the pathogens attack such as *Phytophthora cinnamomi*, together long drought periods, are the main cause of the decline and tree mortality (Brasier, 1996; Ruiz‐Gómez et al., 2018; San‐Eufrasio et al., 2021b; Maldonado-Alconada et al., 2022). For the preservation of these species in the face of imminent climate change that will worsen the situation, urgent measures must be taken, among which biotechnology has a place. The contribution of proteomics to the study of *Q. ilex* has been remarkable, as stated in several works and reviews (**Table S1**).

One of the major limitations reported when working with samples of *Q. ilex* trees from the field is the huge biological variability inter- and intra-population (Jorge et al., 2005; Jorge et al., 2006), to which must be added no-controlled environmental conditions, such as those of field. For this reason, most of the studies of the last decade have been carried out on seedlings, under greenhouse-controlled conditions, introducing a representative number of replicates per experimental condition. Another limitation when working with *Q. ilex* has been the scarce genomic information, such as for other orphan tree species, which forced a long time to work with orthologous sequences (Romero-Rodriguez et al., 2014; Rey et al., 2019). The creation of a reference transcriptome database of *Q. ilex* by Guerrero-Sanchez et al. (2017) markedly improved protein identification success (Gómez-Gálvez et al., 2020; Escandón et al., 2021b). In addition, the integration of multi-omic (transcriptomics, proteomics and metabolomics) data allowed the partial reconstruction of the metabolism of *Q. ilex*, in which TCA cycle was the most represented pathway in the three levels of regulation (López-Hidalgo et al., 2018). Recent advances in genome sequencing have allowed for the first draft of the *Q. ilex* genome (Maldonado-Alconada et al., 2022), which will mean a significant improvement in molecular studies of this species.

Beyond the experimental limitations, the first proteomics works on *Q. ilex* aimed to characterize and catalog Andalusian *Q. ilex* populations and provenances based on the leaf 2-DE profile (Jorge et al., 2005; Jorge et al., 2006), seeds (Valero-Galván et al., 2011) and pollen (Valero-Galván et al., 2012). The topic that has aroused the greatest interest due to the number of publications is the study of the response to biotic and abiotic stresses. Specifically, the response to drought and to the soil pathogen *P. cinnamomi*, the main causes of the decline syndrome, has been the subject of numerous proteomic studies on holm oak. Valero-Galván et al. (2013) observed a reduction of proteins related to ATP synthesis and photosynthesis in seedlings leaves of two *Q. ilex* Andalusian provenances in response to drought by using a gel-based coupled to MALDI-TOF strategy. A decrease in proteins of the carbohydrate metabolism and an increase in ATP synthesis and secondary metabolism were observed in *Q. ilex* seedlings roots in response to water shortage using the same strategy (Simova-Stoilova et al., 2015). When comparing roots and cotyledons, the same authors emphasize the importance of sink-source interaction between root and cotyledon in the time course of stress and recovery (Simova-Stoilova et al., 2018). More recently, a panel of putative markers of tolerance to the drought of *Q. ilex* has been proposed, among which the protease subtilisin and the chaperone GrpE were considered the most promising (San-Eufrasio et al., 2021a). For that, the leaves proteome of seedlings from 4 Andalusian *Q. ilex* populations was analyzed using a shotgun (LC-MSMS) proteomic strategy combined with proteotypic peptides quantification.

Studies of response to *P. cinnamomi* on *Quercus* species are more limited. Sghaier-Hammami et al. (2013) observed an increase of proteins related to starch biosynthesis, glycolysis, and stress-related peroxiredoxin upon inoculation in *Q. ilex* seedlings leaves using 2DE coupled to MALDI-TOF strategy. A shotgun analysis was performed by Saiz-Fernández et al. (2022) using micropropagated clonal *Q. suber* and *Q. variabilis* plants to study the response to *P. cinnamomi. Q. variabilis* displayed a greater upregulation of stress-related proteins in leaves compared to *Q. suber*, namely peroxidases, superoxide dismutases, and glutathione S-transferases, together with proteins related to jasmonic acid metabolism. The authors stated that these differences could be responsible for the higher susceptibility of *Q. suber* to *P. cinnamomi* attack. To our knowledge, the only proteomic work combining drought stress and *P. cinnamomi* inoculation was performed by San-Eufrasio et al. (2021b) in seedlings from two Andalusian *Q. ilex* populations. Using a shotgun proteomics strategy, authors proposed the proteins aldehyde dehydrogenase, glucose-6-phosphate isomerase, 50S ribosomal protein L5, and a-1,4-glucan-protein synthase [UDP-forming] as putative markers for resilience.

The translational potential of proteomics is reflected in recent studies carried out on *Q. ilex* seeds. To understanding the recalcitrant character of these non-orthodox seeds the maturation and germination stages have been studied using different proteomics platforms. Results obtained demonstrated that mature seeds have all the machinery necessary for rapidly resuming metabolic activities and starting the germination process, while post-germination events were similar to that of the orthodox seeds (Romero-Rodríguez et al., 2019; Sghaier‐Hammami et al., 2020). A targeted strategy based on the identification of proteases and proteases inhibitors was carried out using a combination of shotgun and protease activity, giving clues about proteins that may be related to seed quality and viability (Escandón et al., 2022). On the other hand, but not least, proteomics has contributed to the characterization of allergens. The first allergen from *Q. ilex* pollen has been identified by using a targeted proteomics and transcriptomics strategy (Pedrosa et al., 2020), whose interest can be transferred to the pharmaceutical sector. This strategy is being used in the identification of bioactive peptides, probing its nutraceutical value, that will give an added value to holm oak and its use in human nutrition (Maldonado-Alconada et al., 2022).

## 3 Future directions

Proteomics has great potential, constituting priority research for any organism, since the number of protein species differs from the number of genes and transcripts, approaching the phenotype more than the genotype. In the case of forest species, proteomics has been limited by the characteristics of the biological system itself. Therefore, it is imperative to integrate proteomics with other disciplines and omic techniques from a Systems Biology perspective. Future approaches should also consider different perspectives for bridging single organism data to population studies, as well as targeted studies that allow selecting elite genotypes/individuals based on molecular markers.

### 3.1 From single organisms to population-wide studies

Proteomics has been used for studying protein diversity and its cross-functional roles in complex microbial communities isolated from environmental samples (Muth et al., 2016). However, little attention has been paid to understanding protein variability and the molecular events that lead to ecological/environmental adaptation in tree populations. Using proteomics in order to unveil ecological and evolutionary processes pulls the gene-centered approach out and brings a key element into the game: proteins. One of the pioneering studies was carried out by Valero-Galván et al. (2011) in which the biodiversity from ten populations of holm oak distributed throughout the Andalusia region was estimated based on the acorn proteome profile. A similar study was later carried out by Loewe et al. (2018), in which thirty *Pinus taeda* Chilean populations were investigated with the aim of detecting variability across three Chilean macrozones and to provide the molecular basis for conservation purposes in this species. Understanding genetic biodiversity from proteomics may also shed a light on the molecular and phenotype responses of populations to climate change. Small and large range size *Eucalyptus* co-occurring populations were investigated to predict environmental responses upon heatwaves (Aspinwall et al., 2019). Experimental data showed different environmental responses across the populations studied, highlighting the influence of range size and growth temperature in the responses of *Eucalyptus* species. A comprehensive proteomic study of *E. grandis* from six different populations provided evidence of adaptive variation in protein response to temperature extremes at the population level (Maher et al., 2019). This result has a direct impact on conservation biology as it illustrated the importance of taking into consideration the molecular responses to environmental scenarios when elaborating local restoration programs. Those studies do illustrate the potential of population proteomics in order to reveal cryptic diversity and to better represent field responses to environmental changes. However, there is a clear lack in population-wide studies of tree species though they are unequivocally embraced in different ecological niches and represent the most iconic species in the forest biome.

### 3.2 Proteomics as a driver for molecular breeding

Since its conception, proteomics has played a major role in characterizing natural events from a holistic perspective. Organisms, organs, and tissues have been investigated in a large-scale fashion through discovery-driven approaches, in which a massive amount of data is generated. Given the heterogeneous cell information of individual cells, single-cells expression profiling of plant tissues is the only holistic way of generating a deeper understanding of plant developmental processes or environmental adaptation (Clark et al., 2022). From the plant breeding perspective, understanding the molecular mechanisms underlying the adaptation of plants to a specific condition may assist in the selection of plants with genotypes with particular characteristics. The selection of elite genotypes based on molecular markers is a plausible biotechnological approach. Proteomics has contributed greatly to the identification of these markers, which together with other omics disciplines, and after validation both by genomic association and by functional genomic analysis, may accelerate the identification of these genotypes to be used in forest breeding programs. For instance, large-scale proteomics has revealed the molecular regulatory mechanisms of resin yield in *Pinus* plants and allowed the identification of candidate genes for molecular breeding (Li et al., 2022). Proteoforms involved in the Calvin-Benson cycle have been relatively quantified to pinpoint regulatory points in the carbon assimilation pathway in *Eucalyptus* (Marques dos Santos and Balbuena, 2017). A panel of putative molecular markers of tolerance to drought and against *P. cinnamomi* has been proposed on holm oak (San-Eufrasio et al., 2021a; San-Eufrasio et al., 2021b). These studies represent only a fraction of a myriad of similar discovery-driven papers currently available that illustrate the power of proteomics to mine and gather molecular information for plant breeding. Despite all the available toolboxes for genetic transformation and potential targets unveiled by proteomics experiments, there is a clear gap in the use of proteomics to study genetic engineering events in tree species, either by regular discovery-driven approaches (*i.e*. data dependent−DDA or data independent acquisition−DIA) or by targeted data analysis (*i.e.* selected/multiple/parallel reaction monitoring: SRM, MRM, PRM). Besides playing an important role in the selection of gene targets, proteomics in plant species usually reveal a large number of proteins with unknown functions. This phenomenon is intensified in forest trees as annotations from public databases lag their crop’s peer species such as maize and soybean. Therefore, more attention should be paid to genome annotation and sequencing as a way to improve confident gene product identification and quantification. Using proteomics as a scanning method to detect changes in the abundance of proteins to be further characterized as their molecular functions may assist in the improvement of commercially important traits or assist in molecular breeding aiming at climate change mitigation strategies.

## Author contributions

MAC and JVJN: conceptualization. MAC, JP, and TSB: writing-original draft preparation. MC, JP, TSB, and JVJN: writing-review and editing. All authors contributed to the article and approved the submitted version.
